# Cottage Hospital. Keswick

**Published:** 1893-02-11

**Authors:** 


### HOSPITAL CONSTRUCTION.

### COTTAGE HOSPITAL, KESWICK.

The plan of this hospital takes the form of a reversed ⊥. The main entrance being in the centre of the horizontal stroke, the side wings being the wards, and the upright stroke administrative offices. The central part projects 5 ft. 6 in. in front of the wings, and is partly two storeys in height—the remainder being one storey. In the centre of the front is a porch which leads into a vestibule and thence into a large entrance hall. On either side of the vestibule is a ward for one bed. The ward wings each contain a ward for four beds and a nurses' room, the latter being placed between the four bed and the one bed wards, with a window overlooking each. The large wards are 22 ft. by 20 ft., giving a floor area of 110 ft., exclusive of a bay window at the further end.

To each ward is attached a w.c. and a lavatory, with a through-ventilated lobby of approach. No provision appears to be made for cleansing bed-pans, &c.—a somewhat important omission in an otherwise excellent plan.

In the rear building is the matron's sitting-room, with store-room adjoining, staircase to upper floor, w.c. for staff, operation-room, and kitchen offices. The doors of the operation-room, the w.c., and the kitchen are in unpleasantly near neighbourhood, and the operation-room windows are too close to the scullery window. The operation-room is, in fact, unfortunately placed, and without great care in working this arrangemant may, in the future, be the cause of serious mischief.

In a detached building at the back is the mortuary, wash house, coal store, &c. The upper floor over the central part are the bed-rooms for nurses and servants.

A noticeable feature in the exterior is the open verandah, which is attached to each ward.

The plans were designed by Mr. George Dale Oliver, of Carlisle.

